# Multiple endocrine neoplasia 2A with RET mutation p.Cys611Tyr: A case report

**DOI:** 10.1097/MD.0000000000026230

**Published:** 2021-06-04

**Authors:** Yan Li, Ya-qin Tan, Zhi-xiang Tang, Qing-hui Liao, Zhong-qiu Guo, Kang-bao Lai, Rong Wang, Yu-hua Chen

**Affiliations:** Department of Endocrinology and Metabolism, Longgang District People's Hospital of Shenzhen, The Third Affiliated Hospital (Provisional) of The Chinese University of Hong Kong, Longgang central city, Shenzhen, Guangdong, P.R. China.

**Keywords:** c.1832G>A, C611Y, multiple endocrine neoplasia 2A, p.Cys611Tyr, rearranged during transfection protooncogene

## Abstract

**Rationale:**

Multiple endocrine neoplasia 2A (MEN2A) is a rare autosomal-dominant genetic syndrome, frequently misdiagnosed or neglected clinically, resulting in delayed therapy to patients.

**Patient concerns:**

A 47-year-old Chinese male patient underwent laparoscopic right adrenal tumorectomy, and postoperative pathology confirmed the tumor as pheochromocytoma (PHEO). He was readmitted to the department of endocrinology and metabolism due to constant increase in carcinoembryonic antigen (CEA) at 5 months after the operation.

**Diagnosis:**

The patient was confirmed with medullary thyroid carcinoma (MTC), multiple neck lymph node metastasis, and pituitary microadenoma. The p.Cys611Tyr (c.1832G>A, C611Y) mutation was detected. Therefore, he was diagnosed with MEN2A.

**Interventions:**

He underwent total thyroidectomy. The gene-sequencing analysis of his family was conducted, and the C611Y mutation was detected in his daughter.

**Outcomes:**

The level of carcinoembryonic antigen decreased significantly after thyroidectomy in this patient. Long-term follow-up management was conducted. Elevated serum calcitonin and bilateral thyroid nodules were found in his 13-year-old daughter. Thus, MEN2A was highly suspected and she was suggested to undergo total thyroidectomy.

**Conclusion:**

Patients with MEN2A should be screened regularly and managed by a multidisciplinary team.

## 1 Introduction

Multiple endocrine neoplasia (MEN) is characterized by the occurrence of tumors involving ≥2 endocrine glands in a single patient. Up to now, 3 major types of MEN have been recognized and referred to as type 1 (MEN1), type 2A (MEN2A), and type 2B (MEN2B). Multiple endocrine neoplasia 2A (MEN2A) is a rare autosomal-dominant genetic syndrome,^[1]^ first reported by Sipple in 1961^[2]^ and officially named by Steiner 7 years later.^[3]^ Four clinical variants of MEN2A have been recorded in the Revised American Thyroid Association Guidelines for the Management of Medullary Thyroid Carcinoma issued by the American Thyroid Association,^[4]^ including MEN2A, MEN2A with pruritic cutaneous lichen amyloidosis, MEN2A with Hirschsprung disease (HSCR), and familial thyroid medullary carcinoma. The main clinical manifestations of MEN2A are medullary thyroid carcinoma (MTC, 100%), pheochromocytoma (PHEO, 50%), and primary hyperparathyroidism (PHPT, 25%), with an incidence of about 1/30,000.^[5]^ Here, we report a case of MEN2A and conduct a literature review.

## 2 Case presentation

**Figure 1:**
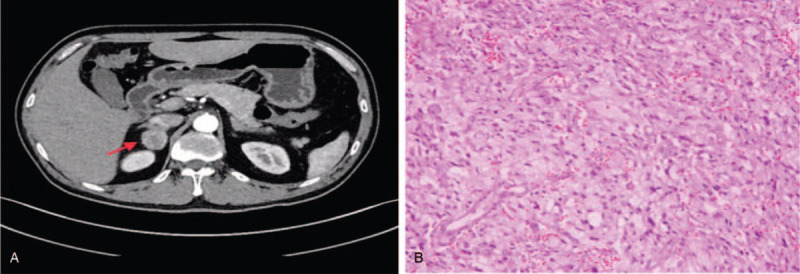
A, Computed tomography of the adrenal glands (preoperative). A 2.6 × 2.8 × 2.4 cm mass was found at the right adrenal gland. Its boundary was clear, and enhancement CT scanning showed it was moderately and heterogeneously enhanced. Figure B, Pathological biopsy of the adrenal glands stained with HE (200×). The tumor cells were mainly large and polygonal in shape, round-to-oval with abundant granular basophilic cytoplasm. The cell nucleuses were round or integral in shape. CT = computed tomography.

**Figure 2:**
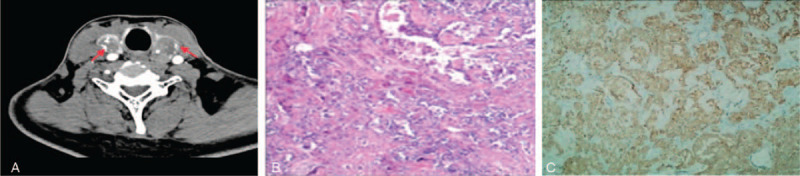
A, Enhanced CT scanning of the thyroid. The thyroid was enlarged, a 2.4 × 2.0 cm round hypodense mass was observed at the left lobar thyroid. The edges were clear, and enhancement CT scanning showed it was heterogeneously enhanced. Several soft tissue masses were found in bilateral carotid space. The larger one was located on the right side, with the size of 3.2 × 3.0 × 4.5 cm. The enhanced scan was homogeneous with slight enhancement. Figure B, Pathological biopsy of MTC stained with HE (100×). Tumor cells were arranged like nests, sheets, islands, which has abundant cytoplasm with light staining or slightly basophilic color, oval nuclei, uniform size, coarse chromatin granules, vaguely visible small nucleoli, and rare mitotic figure. Amyloid deposits were seen in the interstitium. Figure C, The immunohistochemical characteristics of the thyroid (100×), CT+, NSE+, Syn+, CgA+, TG-, Ki67 (5%+), CK+. CT = computed tomography.

**Figure 3:**
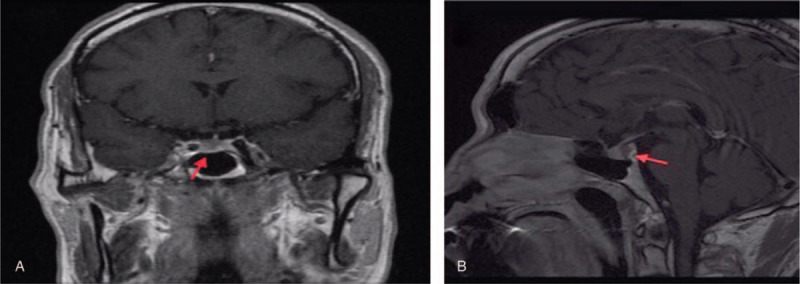
(A, B) Pituitary MRI. An abnormal signal about 0.7 × 0.5 cm was found at the pituitary. The signal intensity was slightly high on T1WI and T2WI. No clear enhancement was found on the enhanced scan. MRI = magnetic resonance imaging; T1WI = weighted imaging; T2WI = T2 weighted imaging.

The patient was a 47-year-old Chinese man, who underwent laparoscopic right adrenal tumorectomy in December 2018. Postoperative pathology confirmed the tumor as the PHEO. Five months after the operation, he was reexamined at the outpatient department and subsequently admitted to the department of endocrinology and metabolism, due to constant increase in carcinoembryonic antigen (CEA). The results of clinical biochemical tests at admission are shown in Table 1. During the reexamination, bilateral thyroid tumor and pituitary microadenoma were detected. The pathologic test revealed squamous epithelial papillomata on the pharynx and adenomatous polyps. Therefore, he underwent total thyroidectomy in May 2019. During the surgery, parathyroid adenomas or hyperplasia was not observed, but bilateral MTC and multiple neck lymph node metastasis were confirmed through postoperative pathology. Combined with the diagnoses of PHEO, MTC, and pituitary microadenoma, he was confirmed with MEN2A. (See Figs. 1–3 for details).

**Table 1:** The results of clinical biochemical tests of the proband.

Clinical biochemical tests of proband			
Items	Preoperative	Postoperative	Reference value
Urinary VMA, mg/24 h	14.10 ↑ (before adrenal sparing surgery)	6.30	≤12.00
Urinary adrenaline, μg/24 h	6.67		0.00–20.00
Urinary noradrenaline, μg/24 h	78.75		0.00–90.00
Urinary dopamine, μg/24 h	108.19		0.00–6000.00
ACTH (08:00), pmol/L	4.67		1.60–13.90
Blood serum cortisol, nmol/L	156.10		133.00–537.00
Urine free cortisol, μg/24 h	38.50		3.50–45.00
ALD, pg/mL	110.00		10.00–160.00 (supine position)
PRA, ng/mL/h	2.34		0.15–2.33 (ordinary diet and supine position)
TSH, μIU/mL	4.59		0.49–4.91
FT3, pg/mL	3.70		2.14–4.21
FT4, ng/dL	0.73		0.59–1.25
Tg, ng/mL	14.55		3.50–77.00
CT, pg/mL	>2000.00 ↑	597.80	0.00–9.52
Serum calcitonin, mmol/L	2.30	2.23	2.00–2.57
PTH, pmol/L	2.89	1.73	1.60–6.90
PCT, ng/mL	82.94 ↑	6.08	0.00–0.05
PRL, ng/mL	11.24		2.64–13.13
LH, μIU/mL	3.80		1.24–8.62
FSH, μIU/mL	8.24		1.27–19.26
TESTO, ng/mL	3.60		1.75–7.81
CEA, ng/ml	731.90 ↑	91.20	<5.09

**Figure 4:**
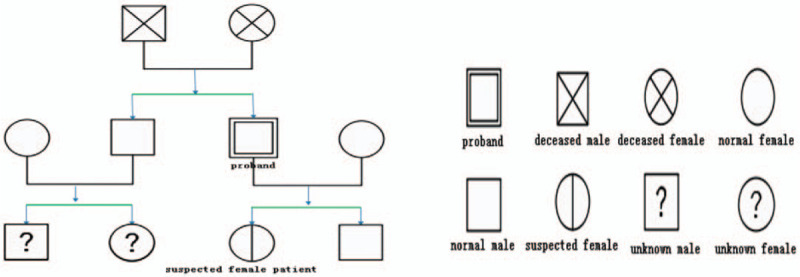
The family tree genealogically. We collected the pedigree members’ clinical data and charted the family tree genealogically.

RET gene sequencing is the gold standard for the diagnosis of MEN, thus the gene-sequencing analyses of this patient and his family were conducted (his brother, his son, his daughter, and himself). The results showed that no RET gene mutation was detected in his brother or his son, but the p.Cys611Tyr (c.1832G>A, C611Y) mutation was detected in his daughter and himself. Since it is one of the pathogenic mutations of MEN2A, we conducted the preliminary screening of his 13-year-old daughter and found bilateral thyroid nodules and elevation in the serum calcitonin level. Thus, she was highly suspected of MEN2A and suggested undergoing total thyroidectomy. Long-term follow-up management was conducted both on this patient and his daughter (Fig. 4).

RET proto-oncogene (rearranged during transfection proto-oncogene) with a total length of 60 kb is the only pathogenic mutation of MEN2A, which is located on the long arm of chromosome 10 (10q11.21) including 21 exons, encoding a RET transmembrane protein composed of 1100 amino acids. This mutation of the RET gene encodes abnormal RET protein, influencing the proliferation, apoptosis, invasion, and metastasis of different tumor cells through the signal pathway, leading to diseases such as MEN2A.^[6–8]^ RET gene mutations have been found in almost all patients with MEN2. The most frequent mutations of the RET gene are point mutations in exons 10 and 11. The distributions of mutational hot spots probably vary across countries, regions, and ethnic populations. The 611 codon is located on exon 10 of the RET gene. The C611Y mutation (the sequence of TGC at the 611 site of the RET gene coding for Cysteine mutates to the sequence of TAC coding for Tyrosine) could cause the formation of ligand-independent RET dimer, automatically activate the downstream signal pathway without ligand, and induce excessive cell proliferation and carcinogenesis.^[9]^

According to the risk of invasive MTC, the RET gene mutations were divided into 4 risk levels by the American Thyroid Association. The C611Y mutation belongs to ATA-b (moderate risk),^[4]^ which is mainly related to MEN2A, familial medullary thyroid carcinoma (FMTC), and MEN2A with HSCR.

## 3 The clinical characteristics of Chinese MEN2A families with the C611Y mutation

### 3.1 Materials and method

Relevant articles from January 2009 to January 2020 were searched in the biomedical databases, including PUBMED, CNKI, WANFANG, and VIP, with the keywords of “Multiple endocrine neoplasia 2A,” “MEN2A,” “RET,” “p.Cys611Tyr,” “c.1832G>A,” and “C611Y.” Four Chinese MEN2A families with the C611Y mutation were retrieved (Table 2).^[10–13]^ One family was eliminated later due to incomplete data.^[13]^ The family data in the case presentation is also listed herein (Table 2: family 4). Fisher exact test, chi-square test, and Bonferroni correction were employed for data analysis. *P* < .05 was considered significant. All analyses were done using SAS 9.4 statistical software (Statistical Analysis System, North Carolina, America).

**Table 2:** Clinical characteristics of Chinese MEN2A families with the C611Y mutation.

Family	Individual	Report time	Gender	Age	Onset form	Complication	Surgery	Adverse outcome	Other treatments
1^[10]^	I.1 proband	2016	F	NA	Bilateral PHEO	Biliteral thyroid adenoma	ASS	—	R, WW
	I.2		F	35	Bilateral PHEO	Bilateral MTC	ASS TT (L)+ST (R) TT (R)+ BiLND	Recurrence and LNM after ST (1 year).	
	I.3		F	42	Bilateral PHEO and bilateral MTC	—	ASS TT+ BiLND	—	—
	I.4		M	18	Gene carrier				WW
2^[11]^	II.1 proband	2017	F	42	Bilateral PHEO and unilateral MTC	—	ASS TT+BiLND	—	—
	II.2		F	NA	Unilateral PHEO and unilateral MTC	—	ASS ST TT+ BiLND (?)	Recurrence	
	II.3		F	65	Unilateral MTC	—	Operation method not mentioned.	—	
	II.4		M	17	Gene carrier				WW
3^[12]^	III.1proband	2018	M	74	Bilateral MTC	—	TT+MBiND	LNM	
	III.2		F	73	Bilateral MTC	—	TT+BiLND	—	
	III.3		M	69	Unilateral PHEO and bilateral MTC	—	ASS (R) TT+BiLND	—	
	III.4		M	61	Bilateral MTC	—	TT+MBiND	LNM	
	III.5		M	30	Bilateral MTC	—	TT (R)+ST (L) TT (L)+MBiND	Recurrence and LNM	
	III.6		F	56	Thyroid adenoma	—	—		R, WW
	III.7		M	42	Bilateral MTC	—	TT (R)+ST (L) TT (L)+MLND	Recurrence and LNM	
	III.8		F	29	Bilateral MTC	—	TT (R)+ST (L) BiLND+MRND	LNM	
	III.9		F	37	Unilateral PHEO	Bilateral MTC CLA	ASS (R) TT+MBiND	LNM	
	III.10		F	35	Bilateral MTC	CLA	ST+MLND TT (R)+MBiND	Recurrence and LNM.	
	III.11		M	42	Clinical suspected MTC				UST
	III.12		F	39	Clinical suspected MTC				UST
	III.13		F	29	Bilateral MTC	—	TT+BiLND	—	
	III.14		F	23	Bilateral MTC	—	TT+BiLND	—	
	III.15		M	20	Gene carrier				WW
	III.16		M	13	Gene carrier				WW
	III.17		M	5	Gene carrier				WW
4	IV.1proband	2020	M	47	Unilateral PHEO	Bilateral MTC	ASS TT+BiLND	LNM	
	IV.2		F	13	Biliteral thyroid adenoma	—		—	R, WW

## 4 Results

### 4.1 The pathogenetic types of the 4 families with MEN2A caused by the C611Y mutation

#### 4.1.1 Penetrance

In the 4 Chinese MEN2A families, 27 people were detected with the C611Y mutation, in which 22 people were diagnosed with MEN2A and the other 5 were mutation carriers, with a total penetrance of 81.48% (22/27). It was noted that there was a significant penetrance difference in sex (*P* < .05). Excluding 4 cases clinically suspected with MTC but not confirmed by pathological biopsy, the penetrance of MEN2A in women was 100% (MTC 100%, PHEO 40.00%), while that in men was only 58.33% (MTC 58.33%, PHEO 15.38%).

#### 4.1.2 Initial symptom

45.45% (10/22) of them were initially diagnosed with MTC, with an average age of 46.10 years (23–74 years). 18.18% (4/22) of the patients had PHEO as the initial symptom, with an average age of 39.67 years (35–47 years). 18.18% (4/22) of them had both PHEO and MTC initially, and their average age at diagnosis was 51.00 years (42–69 years). There was no statistical difference in sex among the above 3 groups.

#### 4.1.3 Final diagnosis

Among the 22 patients with MEN2A, 17 were confirmed with MTC, and 8 were confirmed with PHEO, but no one with PHPT (Table 2). Thus, we conclude that the thyroid gland was more involved in the 4 Chinese MEN2A families, rather than the adrenal gland or parathyroid gland (*P* < .05).

### 4.2 Adverse prognosis of the 4 Chinese MEN2A families due to the C611Y mutation

#### 4.2.1 Recurrence and metastasis

Five out of 17 patients pathologically diagnosed with MTC underwent postoperative recurrence, with an incidence of 29.41% (5/17), and there was no statistically significant difference in sex (*P* > .05). Nine patients had lymph node metastasis of varying severity, accounting for 52.94% (9/17), and there was no significant difference in sex as well (*P* > .05). In addition, there was no adverse prognosis report related to PHEO or PHPT.

#### 4.2.2 Death

No death cases have been reported.

## 5 Conclusion

From 2009 to 2020, scholars reported a total of 4 Chinese MEN2A families with the C611Y mutation. Through a careful literature analysis, the clinical data of 22 patients and 5 gene carriers in the families were extracted and the clinical characteristics were summarized.

The C611Y mutation is one of the pathogenic mutations causing MEN2A. In the 4 Chinese MEN2A families with the C611Y mutation, there was a high genetic penetrance and a significant sex difference. All female patients with the C611Y mutation had MTC, while the penetrance in men was only 58.33%. Thus, we conclude that the thyroid gland was more involved rather than the adrenal gland or parathyroid gland. Besides, we found that MTC was more prevalent as the initial symptom, followed by PHEO. In general, the diagnosis and treatment of PHEO are relatively early for patients with dizziness, heart palpitations, or other similar symptoms. But the early diagnosis of MTC was difficult due to its insidious onset and long-term disease course. No parathyroid involvement was reported in all cases.

Only MTC-related adverse prognosis reports were found in the literature, mainly postoperative recurrence or lymph node metastasis, but metastasis and postoperative recurrence were independent of sex. Fortunately, there was no report of distant metastasis or death.

## 6 Discussion

### 6.1 Discussion of the problems in the management of this case

In this report, the patient was diagnosed with PHEO, MTC, and pituitary microadenoma. Pituitary adenoma was one of the main manifestations of MEN1 (about 40% of MEN1 patients had pituitary adenoma).^[14,15]^ Thus, the patient showed the clinical features of both MEN1 and MEN2A. The tumor spectrum in MEN4 overlapped those of MEN1 and MEN2.^[16,17]^ Should this patient be classified into MEN4? In fact, the incidence of pituitary adenoma was about 741.3/1,000,000,^[18]^ so pituitary tumor was more likely to exist independently of MEN2A in this patient. This could be confirmed by testing the known MEN4 related genes. During the diagnosis and treatment of this patient, he was found with squamous epithelial papillomata on the pharynx and adenomatous polyps. Were these associated with the C611Y mutation?^[19–21]^ More investigations were carried out.

### 6.2 Discussion of the relevant medical literature

In the literature review, there was a significant sex difference in the clinical characteristics of the 4 Chinese MEN2A families with the C611Y mutation, with a penetrance of 58.33% and 100% in male and female patients, respectively. Therefore, regular MTC screening and long-term follow-up management are particularly important for people with C611Y mutation, especially for women. Only MTC-related adverse prognosis reports were found in the literature and it was more prone to postoperative recurrence or lymph node metastasis. So, the selection of operation and treatment of cervical lymph nodes were worth further study. All patients had a recurrence of MTC after subtotal thyroidectomy in the 4 Chinese MEN2A families. Could total thyroidectomy be more beneficial for MTC patients with C611Y mutation? More high-level evidence-based medical studies are expected. The effect of MTC lymph node metastasis or recurrence on the survival rate of MEN2A patients with C611Y mutation, the incidence of surgical complications and adverse outcomes associated with PHEO, and the morbidity of PHPT. In-depth studies are required as unsettled issues still existed.

There are certain limitations in this study. The small number of research subjects may affect our preliminary conclusions. In the future, it is necessary to enlarge the sample size.

### 6.3 The lessons of this case report

It was difficult to make an accurate diagnosis of MEN2A in clinical since its incidence in population was rather low and its clinical symptom was not representative. As a result, the clinical data of the proband (Table 2: IV 1) related to this report was missed diagnosis in December 2018. Therefore, the physicians should be trained to obtain more knowledge about MEN2A, to improve the long-term disease-free survival rate of patients. The detection of the RET gene should be conducted as early as possible to avoid misdiagnosis or missed diagnosis.^[22–24]^

Furthermore, MEN2A should be highly suspected in his daughter (Table 2: IV 2) presenting high serum calcitonin level, bilateral thyroid nodules, and C611Y mutation. But their families rejected our surgical advice to this girl. We should advance communication between patient and doctor. Patients and their families should be aware of the risk of MEN2A and the importance of prevention, early diagnosis, and early normalized treatment. The children in their families should receive age-appropriate information in a friendly and caring way. Psychological support and genetic counseling should be provided throughout the entire process.^[25]^ In conclusion, MEN2A patients should be screened regularly and managed by a multidisciplinary team.

## Author contributions

**Conceptualization:** Yan Li, Ya-qin Tan, Yu hua Chen.

**Data curation:** Yan Li, Ya-qin Tan, Zhi-xiang Tang, Qing-hui Liao, Zhong-qiu Guo, Kang-bao Lai, Rong Wang, Yu hua Chen.

**Formal analysis:** Yan Li, Yu hua Chen.

**Funding acquisition:** Yu hua Chen.

**Investigation:** Yan Li, Ya-qin Tan, Zhi-xiang Tang, Qing-hui Liao, Zhong-qiu Guo, Kang-bao Lai, Rong Wang, Yu hua Chen.

**Methodology:** Yan Li, Ya-qin Tan, Zhi-xiang Tang, Qing-hui Liao, Zhong-qiu Guo, Kang-bao Lai, Rong Wang, Yu hua Chen.

**Project administration:** Yan Li, Ya-qin Tan, Yu hua Chen.

**Resources:** Yan Li, Ya-qin Tan, Zhi-xiang Tang, Yu hua Chen.

**Software:** Qing-hui Liao, Zhong-qiu Guo, Kang-bao Lai, Rong Wang.

**Supervision:** Qing-hui Liao, Zhong-qiu Guo, Kang-bao Lai, Rong Wang.

**Validation:** Yan Li, Ya-qin Tan, Yu hua Chen.

**Visualization:** Zhi-xiang Tang, Qing-hui Liao, Zhong-qiu Guo, Kang-bao Lai, Rong Wang.

**Writing – original draft:** Yan Li.

**Writing – review & editing:** Yan Li, Ya-qin Tan, Zhi-xiang Tang, Qing-hui Liao, Zhong-qiu Guo, Kang-bao Lai, Rong Wang, Yu hua Chen.
